# Microbiota profiles and intestinal immunity in Bermuda feral chickens: A comparison with commercial broilers

**DOI:** 10.1016/j.psj.2026.107440

**Published:** 2026-07-16

**Authors:** H.W. Kim, R.M. Hayashi, C. Mendez-Garcia, E. Gering, I. Cann, T.G. Rehberger, A.H. Smith, E. Santin, R.I. Mackie

**Affiliations:** a Department of Animal Sciences, University of Illinois Urbana-Champaign, Urbana, IL 61801, USA; b Carle R. Woese Institute for Genomic Biology, University of Illinois Urbana-Champaign, Urbana, IL 61801, USA; c Laboratório de Microbiologia e Ornitopatologia, Universidade Federal do Paraná, Brazil; d Kellogg Biological Station, Michigan State University, Hickory Corners, MI 49060, USA; e Department of Biological Sciences, Nova Southeastern University, Fort Lauderdale, FL 33328, USA; f Arm & Hammer Animal and Food Production, Church & Dwight, Waukesha, WI 53186, USA

**Keywords:** Feral chickens, Microbiota diversity, Toll-like receptors, Innate immunity, Intestinal health

## Abstract

Domestication for production and captive rearing may alter the chicken gut microbiome and compromise immune function in modern broiler chickens. In this study, we compared microbiota, Toll-like receptor (TLR) gene expression, and intestinal health between feral chickens from Bermuda (BFC, *n* = 21) and commercial Cobb 500 broilers (BC, *n* = 12). Microbiota analysis showed that feral chickens harbored greater microbial diversity with higher proportions of Gram-negative bacteria in BFC (31%) vs. BC (8.2%). RT-qPCR analysis, followed by ANOVA and Tukey’s test, revealed higher ileal expression of TLR in BFC and hepatic expression in BC (*P* < 0.05) indicating enhanced mucosal innate immunity in feral chickens and systemic immune activation in broiler chickens, respectively. The upregulation of ileal TLR4 and TLR5 in feral chickens correlated with Gram-negative and flagellated Proteobacteria, respectively. Histological evaluation showed higher Intestinal Scoring Index (ISI) scores in BFC (Kruskal-Wallis test, *P* < 0.05) with increased lamina propria thickness, goblet cell proliferation, and a robust mucosal inflammatory response, while BC showed minimal mucosal inflammation but significant hepatic lymphocytic aggregation and congestion (*P* < 0.05). These findings suggest that feralization and free-living conditions are associated with a high mucosal immune surveillance that effectively limits systemic antigen translocation, while commercial broilers show reduced intestinal immune activation and increased susceptibility to hepatic inflammation. This indicates that selection for intensive production may compromise gut barrier function and shift the site of immune activation from the mucosa to the liver.

## Introduction

Chickens are among the most efficient agricultural animal species for converting feed into lean meat. The modern poultry industry faces strong economic pressure to maximize growth rate and feed conversion efficiency. As a result, selective breeding has focused on traits related to rapid growth and productivity based on genetic growth characteristics, balanced diets, optimized housing condition, floor litter, and controlled stocking density (Tallentire et al., 2016). This process, while effective for production, can unintentionally reduce immune competence and increase disease susceptibility.

Feralization occurs when domesticated animals escape human control and adapt to the wild. It removes artificial selection pressures and restores natural and sexual selection, and this can impact both the gene pools and traits of feral taxa (Gering et al., 2024). Studying feral chickens, therefore, provides a valuable pathway to understanding how long-term domestication and management affect physiology and host-related microbial communities.

Bermuda supports a large self-sustaining population of feral chickens that have been living in the wild since at least the mid-1980s. Based on preliminary genetic and morphological analyses, these birds appear to be an admixed flock originating from several breeds that are popular sources for meat and egg production in the Western hemisphere. Our ad hoc qualitative observations of gut content in Bermuda feral chickens (BFC) suggest a highly variable diet including locally-occurring invertebrates (e.g., snails and insects), local ornamental and/or natural vegetation (e.g., seeds and shoots) and garbage from Bermuda households and businesses. Since BFC has persisted without human management or artificial selection for several decades, it represents a rare and valuable opportunity to examine how sustained exposure to a diverse and unmanaged microbial environment shapes host immune function.

In this study, we investigated microbiota profiles of feral and commercial chickens by using 16S rRNA amplicon sequencing; we then characterized innate immune responses by analyzing Toll-like receptor (TLR) gene expression in the liver and ileum using RT-qPCR. Feral (formerly domesticated, wild-living) chickens from Bermuda were compared with commercial broiler chickens (BC). We also evaluated intestinal health through histological and molecular analyses. Our overall findings demonstrate the immunological consequences of gut microbial shifts during feralization. They suggest the possibility that exposure to more natural environments could enhance microbial and immunological resistance in cultivated chickens ultimately supporting healthier and safer poultry production.

## Materials and methods

### Ethical statement

Collection and export of samples derived from feral chickens was approved by the Bermuda Department of Environment and Natural Resources in support of the Bermuda Biodiversity Project. In addition, this study was performed in accordance with the recommendations in the Guide for the Care and Use of Laboratory Animals of the National Institutes of Health. The protocol was approved by the Institutional Animal Care and Use Committee of the Michigan State University under permit number 06/17-093-00.

### Animals

A total of 21 feral chickens from St. George’s Island, Bermuda and 12 broiler chickens (BC) from the Poultry Farm at University of Illinois Urbana-Champaign, were investigated. All feral birds were harvested from the area surrounding St. George’s Island, Bermuda, and are therefore the outcome of natural environmental conditions, without human control or interference, and termed Bermuda feral chickens (BFC). BFC was selected because it represents one of the long-established feral chicken populations with minimal human intervention, which makes it an ideal model for studying feral chickens. Sample sizes were, however, constrained by the logistical and ethical limitations of sampling wild chickens during the approved collection permit. We also sampled 12 Cobb 500 male broiler chickens from the Poultry Farm, Department of Animal Sciences, University of Illinois (UIUC), raised in the same house and provided with food and water *ad libitum*. The detailed diets from the two evaluated groups are presented in Table S1.

### Genomic DNA extraction and amplification of 16S rRNA gene sequences

Ileal and cecal luminal contents were obtained, frozen in liquid nitrogen and stored at −80°C until analysis. Samples were subjected to DNA extraction using the Power Soil DNA Extraction Kit (Mo Bio Laboratories, Inc., Carlsbad, CA). Amplification of 16S rRNA gene sequences was conducted at the University of Illinois Urbana-Champaign Biotech Center using the Fluidigm system, which permits parallel amplification of a specific region from a target gene prior to high throughput sequencing. Amplicon libraries for the V4 region of the 16S rRNA gene were generated using the primer pair 515F (5´GTGYCAGCMGCCGCGGTAA 3´) and 806R (5´ GGACTACNVGGGTWTCTAAT 3´), delivering an amplified region of 292 base pairs. Sequencing of amplicons was performed using the Illumina MiSeq V3 platform with paired read (2 × 300) sequencing to ensure recovery of the integral V4 region. Resulting amplicon sequences were processed using the QIIME pipeline described in the supplementary materials – Bioinformatic analysis of sequencing results. Alpha diversity was assessed using phylogenetic diversity (PD whole tree), the Chao1 richness estimator, and observed operational taxonomic unites (OTUs) calculated in QIIME. Relative abundance of microbial taxa was also compared between BFC and BC groups.

### RNA extraction and reverse transcription (RT)-PCR

Samples of ileal and liver material from BFC and BC were collected and RNA extracted by TriZol (MBI Fermentas, MD, USA) according to the manufacturer’s instructions. To avoid the possible traces of genomic DNA, 5 mg of each RNA sample was incubated at 37°C for 10 min with 5 U of RNase free DNase. DNase was then inactivated by incubation at 65°C for 10 min. Subsequently, total RNA (5 mg) from each sample was reverse transcribed into cDNA by using the RevertAid First strand cDNA synthesis kit (MBI Fermentas, MD, USA) according to the manufacturer’s instructions. The resultant cDNA was stored frozen at −20°C until further analysis.

### Relative quantification of toll-like receptors mRNA by real time PCR

The relative expression levels of TLR mRNA (TLR1, TLR2, TLR3, TLR4, TLR5, TLR15 and TLR21) from BFC and BC were quantified by real time PCR performed in Mx3000P system (Stratagene, CA, USA). Each reaction was carried out in triplicates in a total volume of 25 mL containing 1 × Maxima SYBR Green/ROX qPCR master mix (MBI Fermentas, MD, USA), 10 pmol concentration of specific primers (Table S2), and 1 mL of cDNA template (100 ng/mL). PCR cycling conditions were as follows: 95°C for 10 min followed by 40 cycles of denaturation at 94°C for 30 s, annealing at 55–60°C for 30 s, and extension at 72°C for 30 s. In each PCR amplification, no template control was included to check contamination of master mix. Non-reverse transcribed RNA (10 ng) of each sample was used instead of cDNA to check contamination of samples with gDNA. Failure of the amplification confirmed the purity of the sample. To assess the efficiency of primers, standard curves for each primer pair were generated using serially diluted transcribed RNA samples. PCR efficiency was calculated from the slope of standard curves. The resulting threshold cycle [Ct, a fractional PCR cycle number at which the change in reporter dye (∆Rn) passes the significant threshold] values were normalized to the endogenous control, beta actin (∆CT = Ct value of target gene – Ct value of beta actin).

### Evaluation of intestinal health – histology by ISI (I See Inside) methodology

Samples of ileum and liver from BFC and BC were collected and further microscopic evaluation of the gastrointestinal tract was performed using the ISI Methodology (“I See Inside”; Pat. INPI-BR1020150036019) as published by Kraieski et al. (2017). Briefly, this methodology was developed based on a numeric score of histological alterations. For each alteration observed during microscopic analysis, an impact factor (IF) was defined according to its importance in affecting organ functional capacity based on previous knowledge of literature and background research (e.g. necrosis has the highest IF because the functional capacity of affected cells is totally lost). The IF ranges from 1 to 3, where 3 represents an IF of the greatest significance in terms of the organ function. In addition, the extent of each alteration (intensity or observed frequency compared to non-affected tissue) was evaluated per field (liver) or per villi (intestine) and scored ranging from 0 to 3. To reach the final ISI value, the IF of each alteration was multiplied by the respective score number, and the results of all alterations were summed.

### Statistical analysis

All statistical analyses were performed with Statistics software version 9.0. Data were evaluated by the Shapiro-Wilk normality test. Parametric data were subjected to analysis of variance (ANOVA) and Tukey's test to establish differences among treatment means. Nonparametric data were submitted to the Kruskal-Wallis test at a 5% probability value. Differences in diversity metrics and relative abundance of microbial taxa between BFC and BC were evaluated using Tukey’s test.

## Results and discussion

### Microbiome diversity and composition

**Fig. 1:**
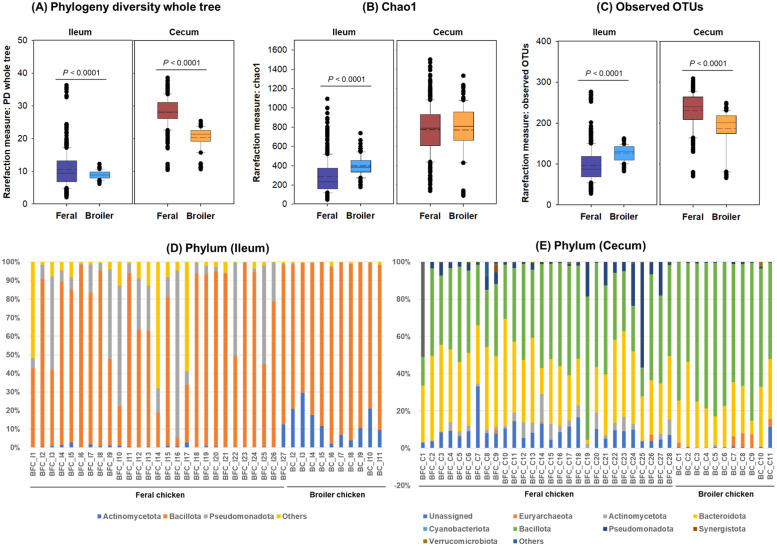
Box plots showing the alpha diversity indices including (A) phylogeny diversity whole tree, (B) chao 1, and (C) observed OTUs in microbiomes of ileal and cecal samples in feral and broiler chickens. Squares indicates the interquartile range for each data point, and the solid and dotted line denote the median and mean values, respectively. The error bars indicate the 10th and 90th percentiles, and the black circles indicate outliers. Statistical analysis was done by Tukey test. Relative abundance of major microbial taxa in the (D) ileum and (E) cecum of chickens sampled at the phylum level.

According to our sequencing data analysis, feral chickens showed greater microbial diversity compared to broiler chickens (Figure 1). In the cecum, alpha diversity metrics (PD whole tree and observed OTUs) were higher in feral chickens than in broiler chickens (*P* < 0.0001). In the ileum, however, broiler chickens showed slightly higher Chao1 (total species richness) and observed OTUs (actual number of taxonomic unites) (*P* < 0.0001) despite lower phylogenetic diversity. We further assessed ß-diversity based on unweighted UniFrac distances, visualized by Principal Coordinate Analysis (PCoA) (Figure S1). Feral and broiler chickens formed clearly separated clusters in both ileum and cecum (*P* < 0.05).

At the phylum level (Figure S2), the ileal microbiome of both groups was largely dominated by Bacillota. Notable differences between groups were that feral chickens showed a higher relative abundance of Pseudomonadota in the ileum (BFC, 21.4% vs. BC, 0.17%, *P* < 0.05), while broiler chickens were characterized by Actinomycetota (13.3%). In the cecum, feral chickens again showed a higher relative abundance of Pseudomonadota (*P* < 0.05). These results contrast with Yadav et al. (2021), who reported higher cecal microbial diversity in pasture-raised chickens compared to Hawaiian feral chickens. However, that study compared 5 week-old domestic birds with much older feral chickens and this may have resulted in the diversity differences.

At the genus level (Figure S3), BC showed significantly higher abundance of several taxa in the ileum (*P* < 0.05), including *Bacillus, Staphylococcus*, and *Turicibacter*, and in the cecum, including *Methanobrevibacter, Blutia, Faecalibacterium*, and *Oscillospira* (*P* < 0.05). Several of these taxa have been associated with gut health and productivity in commercial poultry. For example, *Methanobrevibacter* removes excess bowel hydrogen, thereby improving microbial fermentation and energy extraction.

### TLR expression patterns in the ileum and liver

**Fig. 2:**
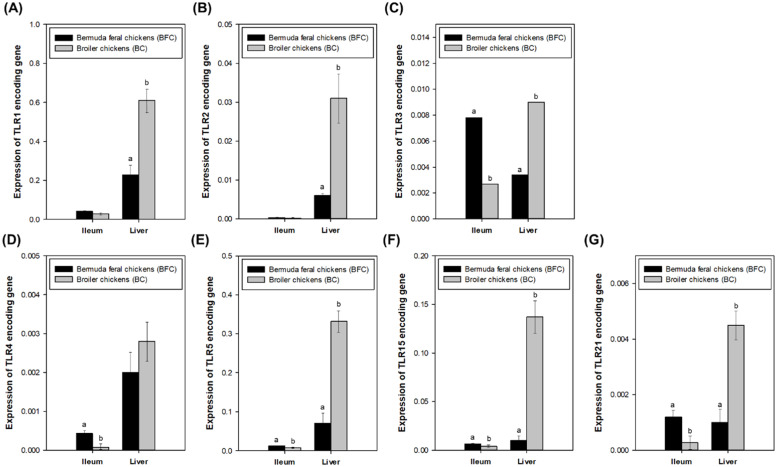
Relative expression of Toll-like receptor (TLR) encoding genes in the ileum and liver of the Bermuda feral chickens (BFC) and broiler chickens (BC). Total of seven TLR encoding genes were analyzed: (A) TLR1, (B) TLR2, (C) TLR3, (D) TLR4, (E) TLR5, (F) TLR15, and (G) TLR21.

The expression analysis of TLR genes showed clear patterns among chicken samples (Figure 2). In the ileum, BFC showed a significant upregulation of TLR3, TLR4, TLR5, TLR15, and TLR21 compared to BC (*P* < 0.05). This indicates a higher activation of mucosal innate immunity in feral chickens, which might reflect adaptation to microbial rich environments. Slightly higher ileal TLR4 and TLR5 expressions in BFC (*P* < 0.1) may reflect subtle microbial differences between habitats and/or divergence in feral gene pools (Gering et al., 2024).

In contrast to the ileum, BC showed higher hepatic expressions of TLR1, TLR2, TLR3, TLR5, TLR15, and TLR21 (*P* < 0.05) (Figure 2). This may reflect reduced intestinal barrier function in BC that allows microbes or microbial products to cross the gut barrier into systemic organs like liver (Wiest and Garcia-Tsao, 2005). Once bacterial components reach the liver, Kupffer cells - the resident macrophages - detect them via TLRs triggering hepatic inflammation (Hammerich and Tacke, 2023).

### Association of ileal TLR expression with microbiota diversity

The differential ileal TLR expression observed between the two groups can be linked to specific differences in microbiota composition. Particularly, TLR4, which recognizes lipopolysaccharides of Gram-negative bacteria (Miller et al., 2005) was upregulated in BFC (*P* < 0.05). This aligns with the higher proportion of Gram-negative taxa observed in BFC (31%) compared to BC (8.2%). TLR5, which detects bacterial flagellin and associated with gut mucosal barrier breakdown (Cullender et al., 2013), was also upregulated in feral groups. This is consistent with an enrichment of flagellated Pseudomonadota (*P* < 0.05) in feral chicken groups, a phylum in which many members possess flagella for motility and may therefore contribute to the elevated TLR5 expression observed in this group. Increased TLR activity supports epithelial integrity and immune tolerance. The observed upregulation of mucosal TLRs in feral chickens, therefore, might reflect controlled immune mechanism that supports resilience against environmental microbes that enter with the diet.

### Histological alterations in the ileum and liver

Microscopic evaluation using the Intestinal Scoring Index (ISI) showed distinct histological differences among groups (Figure S4). BFC showed higher ISI scores than BC (*P* < 0.05, Figure S4A). Feral chickens showed marked expansion of the lamina propria thickness (LPT) with dense mixed inflammatory infiltrates (MIILP), vascular congestion (CG), and increased goblet cell numbers (Figure S4C). In contrast, broiler chickens showed relatively preserved villus architecture with milder inflammatory cell infiltration (Figure S4D). These observations suggest that exposure to diverse microbial environments induces mucosal inflammation in feral chickens. This might represent an active defense strategy in response to high antigenic exposure, which maintains a functional barrier necessary to survive in a pathogen-rich environment. Diets of feral chickens are composed of insects, seeds, vegetation, and anthropogenic waste that might drive both microbial and immune variation. As the exposure to diverse microbial-associated molecular patterns activates pattern-recognition receptors such as TLRs, increased ileal TLR expression and histological immune activation in feral chickens appear to be related to ecological adaptation.

In addition to ileal alterations, histological examination of the liver also revealed distinct differences between groups. It confirmed lymphocytic aggregation and congestion in BC livers (*P* < 0.05; Figure S4B). In contrast, feral livers showed lower ISI inflammation scores. BFC showed mild pericholangitis (PER) and hydropic degeneration (Figure S4E), whereas BC livers showed more lymphoid aggregates (LA) and congestion (CG, Figure S4F). This contrast between high ileal inflammation and low hepatic activation in BFC suggests an effective compartmentalization of the immune response. The robust mucosal defense in the gut of feral chickens successfully prevented systemic translocation of pathogens to the liver despite the evident tissue infiltration.

### Immunological and ecological implications of feralization

The contrasting TLR profiles of feral and broiler chickens demonstrate the immunological outcomes of domestication and feralization. Feralization (Gering et al., 2024) allows natural selection to act on traits essential for survival in variable environments, and this likely drives evolution in both host-associated microbial communities and host traits that shape their assembly. In feral chickens, prolonged exposure to fluctuating diets, pathogens, and environmental stress appears to have reactivated innate immune pathways. In contrast, broiler chickens have been optimized for growth, feed conversion, and carcass yield (Tallentire et al., 2016). While these traits improved productivity, they also reduced genetic and microbial diversity that compromises immune response. Downregulation of intestinal TLRs in BC suggests a weakened mucosal sensing capacity and greater susceptibility to infection under non-controlled conditions. These findings support the earlier reports that selection for production can affect physiological traits from immunity to growth (Rauw et al., 1998). Reduced microbial diversity in broilers may further hinder immune maturation, as a diverse microbiota is important for immune development and regulation (Yegani and Korver, 2008).

Exposure to a broad microbial community activates the immune system fostering tolerance to commensals while maintaining defense against pathogens. Increased ileal TLR expression in feral chickens, therefore, reflects an evolved adaptation to microbial coexistence. However, diversity and inflammation interact in complex ways. While a diverse microbiota can prevent pathogen colonization, excessive TLR activation can also cause chronic inflammation and tissue remodeling. This dynamic was evident in BFC, where strong TLR expression coincided with moderate inflammation. Inflammation may also alter microbial ecology in a similar way to mammalian systems (Winter et al., 2010). Thus, maintaining immune balance is critical for gut homeostasis.

### Implications for pathogen ecology

This study focused on individual-level variation, testing how the ancestry and environment of focal animals predict differences in gut microbiomes and immune-related traits. However, our findings may also have implications for pathogen dynamics across coupled domestic, human-modified, and wild systems. Feral animals can play complex roles in the ecology and evolution of diseases transmitted among domesticated animals, wildlife, and human populations (Gering et al., 2024). Feral and backyard chickens are widespread, often occur near both commercial poultry and human communities, and can harbor zoonotic and agriculturally important pathogens. Their contributions to pathogen transmission may depend partly on immune and physiological traits shaped by domestication, feralization, and local environmental conditions.

Our finding that BFC and BC differ in their microbiota, intestinal physiology, and expression of immune-related genes therefore suggests that studies using domesticated *Gallus gallus* may not fully predict pathogen dynamics involving feral populations. For example, stronger mucosal protection could reduce pathogen burden and transmission, whereas greater disease tolerance could allow infected animals to remain active and shed pathogens for longer. Because the present study did not measure infection, shedding, or disease outcomes, the net consequences of the observed differences remain uncertain. Nevertheless, these results identify intestinal phenotype, microbiome composition, local environment, and host ancestry as potentially important sources of variation to consider when extrapolating disease studies from domesticated chickens to feral populations.

Feral chickens show greater inter-individual variance in age, microhabitat, diet, and genetic background compared to inbred farm cohorts raised in a common environment. We, therefore, prioritized sampling depth in the feral group to reduce the influence of outliers on inferred group-wide patterns. The number of birds available for collection was limited by the restricted access to this wild population during the approved collection period. In contrast, the Cobb 500 broiler chickens are more uniform and homogenous that sample size here was determined by the number of tissue and microbiome samples that passed quality control. As the broiler group was included as a reference for comparison, we believe this imbalance does not compromise the main findings. However, future studies with larger sample sizes and deeper analysis of immune response would help further validate these findings.

## Conclusions

The findings in this study show that feralization reshapes the chicken immune system. Feral chickens showed increased intestinal immune activation, functional containment of luminal antigens resulting in reduced hepatic inflammation. This indicates a more balanced systemic immunity. In contrast, broiler chickens showed suppressed gut immunity and increased hepatic activation, which is consistent with increased immune vulnerability. This imbalance in broilers may result from selection for rapid growth under low-microbial conditions. Such reduced adaptability increases dependence on antibiotics and strict hygiene. In contrast, moderate immune activation in feral chickens may promote disease resistance by sustaining mucosal health. These results suggest that controlled reintroduction of microbial diversity or microbiota-based interventions may help restore immune balance. Beyond that, these immune and microbiome differences between feral and domesticated chickens may also shape their relative roles in pathogen transmission across domestic, wild, and human-modified systems. Future research, therefore, should clarify the functional relationships among microbiome composition, TLR signaling and its downstream effectors, and immune performance in poultry.

## Disclosures

The authors declare that they have no known competing financial interests or personal relationships that could have appeared to influence the work reported in this paper.

## Appendix Supplementary materials

Image, application 1

## Footnotes

Supplementary material associated with this article can be found, in the online version, at doi:10.1016/j.psj.2026.107440.
