# Supplementary material for: Microbiota profiles and intestinal immunity in Bermuda feral chickens: A comparison with commercial broilers
Source: Poult Sci. 2026 Jul 16;105(10):107440. doi: 10.1016/j.psj.2026.107440 (PMC13393662; doi:10.1016/j.psj.2026.107440)
Supplement: Supplementary file 1 [file mmc1.docx]

**Supplementary materials**

**Bioinformatic analysis of sequencing results**

The raw paired-reads were quality checked with FastQC, trimmed if necessary using the FASTX-toolkit (<http://hannonlab.cshl.edu/fastx_toolkit/>), and paired merger with PEAR (Zhang et al. 2014). Resulting amplicons were processed using the QIIME pipeline (Caporaso et al. 2011). Reads with quality scores under 25 and chimeric sequences predicted with usearch (Edgar, 2010) were excluded from following analyses. Resulting quality-controlled sequences were binned into operational taxonomic units (OTUs) at a 97% similarity cutoff (Kunin et al. 2010) using uclust (Edgar, 2010). We used the *open reference* OTU picking method, where reads are clustered against the reference database collection, and any unmatched reads are subsequently clustered *de novo*. The cluster seeds were used as representative sequences. These non-chimeric representatives were aligned with the PyNAST algorithm (Caporaso et al. 2011) using as reference the Greengenes core set alignment (DeSantis et al. 2006). Taxonomy assignations were inferred through comparisons with both the RDP and BLASTn databases. Rarefaction analysis was performed to remove the heterogeneity of the number of sequences per sample prior to calculation of alpha and beta diversity statistics. Alpha and beta diversity metrics were calculated using QIIME.

Zhang, J., K. Kobert, T. Flouri, and A. Stamatakis. 2014. PEAR: A fast and accurate Illumina paired-end read merger. *Bioinformatics* 30:614–620.

Caporaso, J. G., J. Kuczynski, J. Stombaugh, et al. 2011. QIIME allows analysis of high-throughput community sequencing data. *Nat. Methods* 7:335–336.

Edgar, R. C. 2010. Search and clustering orders of magnitude faster than BLAST. *Bioinformatics* 26:2460–2461.

Kunin, V., A. Engelbrektson, H. Ochman, and P. Hugenholtz. 2010. Wrinkles in the rare biosphere: Pyrosequencing errors can lead to artificial inflation of diversity estimates. *Environ. Microbiol.* 12:118–123.

DeSantis, T. Z., P. Hugenholtz, N. Larsen, et al. 2006. Greengenes, a chimera-checked 16S rRNA gene database and workbench compatible with ARB. *Appl. Environ. Microbiol.* 72:5069–5072.

**Supplementary comparison with a Hawaiian feral chicken cohort**

To determine if similar immune adaptations occur in feral chickens from other regions, we additionally analyzed feral chickens from Hawaii as a supplementary cohort. The capture and sampling of feral chickens was approved by the University of Hawaii Center for Tropical and Agricultural Research Station. Differences between BFC and HFC were minor but had similar trends; thus, feralization might induce immune adaptations regardless of geographic origin. Slightly higher ileal TLR4 and TLR5 expressions in BFC (*P* < 0.1) may result from subtle microbial differences between Hawaii and Bermuda habitats, and/or from genetic divergence in feral gene pools (Gering et al. 2024).

**Table S1.** Information on chicken samples collected in this study

| **Groups** | **Origin** | **Feed composition** | **Sample number**  **(Cecum)** | **Sample number**  **(Ileum)** |
| --- | --- | --- | --- | --- |
| **Feral chickens** |  |  |  |  |
| Bermuda Feral Chickens (BFC) | St. George (Bermuda) | Invertebrates, seeds, shoots, household and business garbage | BFC_C1 - 28 | BFC_I1 - 27 |
| Broiler Chickens (BC) | Poultry Farm (University of Illinois at Urbana-Champaign) | Cereals (wheat, corn),  protein flour (soy,  sunflower), vegetables  oils (soy), mineral.  This diet contained no antibiotics | BC_C1 - 11 | BC_I2 - 11 |

**Table S2**. Sequence of primers used in RT-PCR

| Gene | Ligand | Forward primer (5’–3’) | Reverse primer (5’–3’) |
| --- | --- | --- | --- |
| TLR1 | Triacylated lipopeptides | AGTCCATCTTTGTGTTGTCGCC | ATTGGCTCCAGCAAGATCAGG |
| TLR2 | Diacylated lipopeptides | GATTGTGGACAACATCATTGACTC | AGAGCTGCTTTCAAGTTTTCCC |
| TLR3 | dsRNA | TCAGTACATTTGTAACACCCCGCC | GGCGTCATAATCAAACACTCC |
| TLR4 | LPS | AGTCTGAAATTGCTGAGCTCAAAT | GCGACGTTAAGCCATGGAAG |
| TLR5 | Flagellin | CCTTGTGCTTTGAGGAACGAGA | CACCCATCTTTGAGAAACTGCC |
| TLR15 | Protease | TGCCCCTCCCACTGCTGTCCACT | AAAGGTGCCTTGACATCCT |
| TLR21 | DNA | GTTCTCTCTCCCAGTTTTGTAAATAGC | GTGGTTCATTGGTTGTTTTTAGGAC |

**
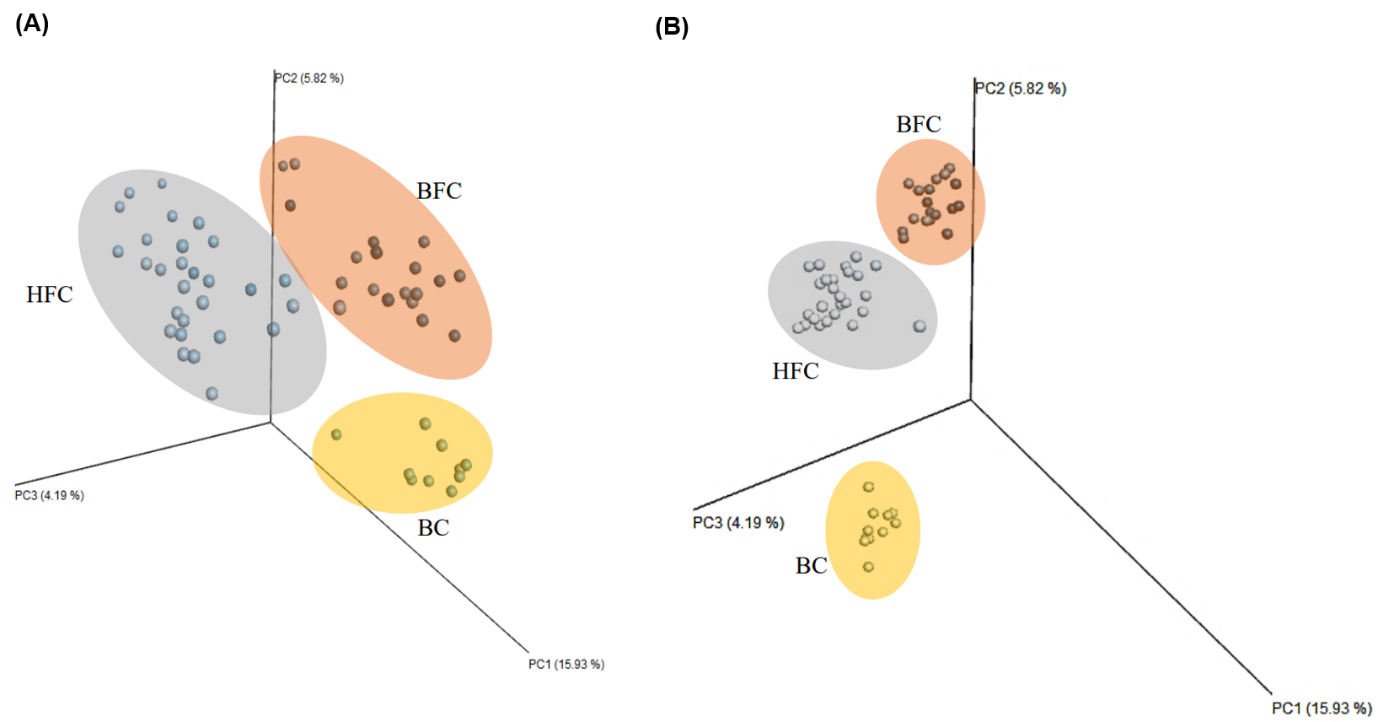
Figure S1.** Weighted UniFrac PCoA plots showing beta diversity analysis among groups. Individual chicken samples of (A) cecum and (B) ileum were analyzed by origin of the samples: Bermuda feral chicken (BFC, orange), Hawaii feral chicken (HFC, grey), and Urbana broiler chicken (BC, yellow).

**
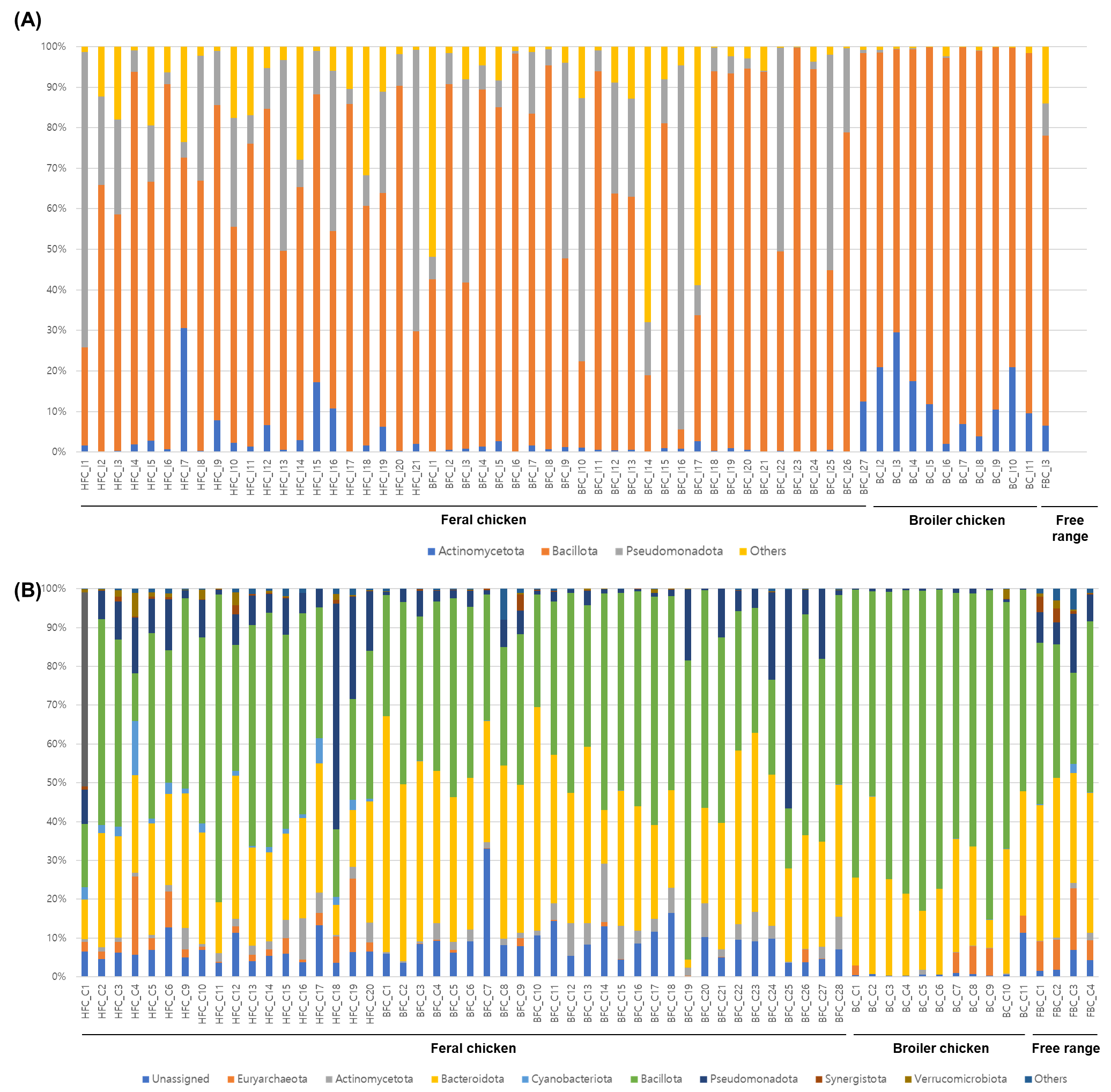
**

**Figure S2.** Relative abundance of major microbial taxa in (A) ileum and (B) cecum of chickens sampled at the phylum level.

**
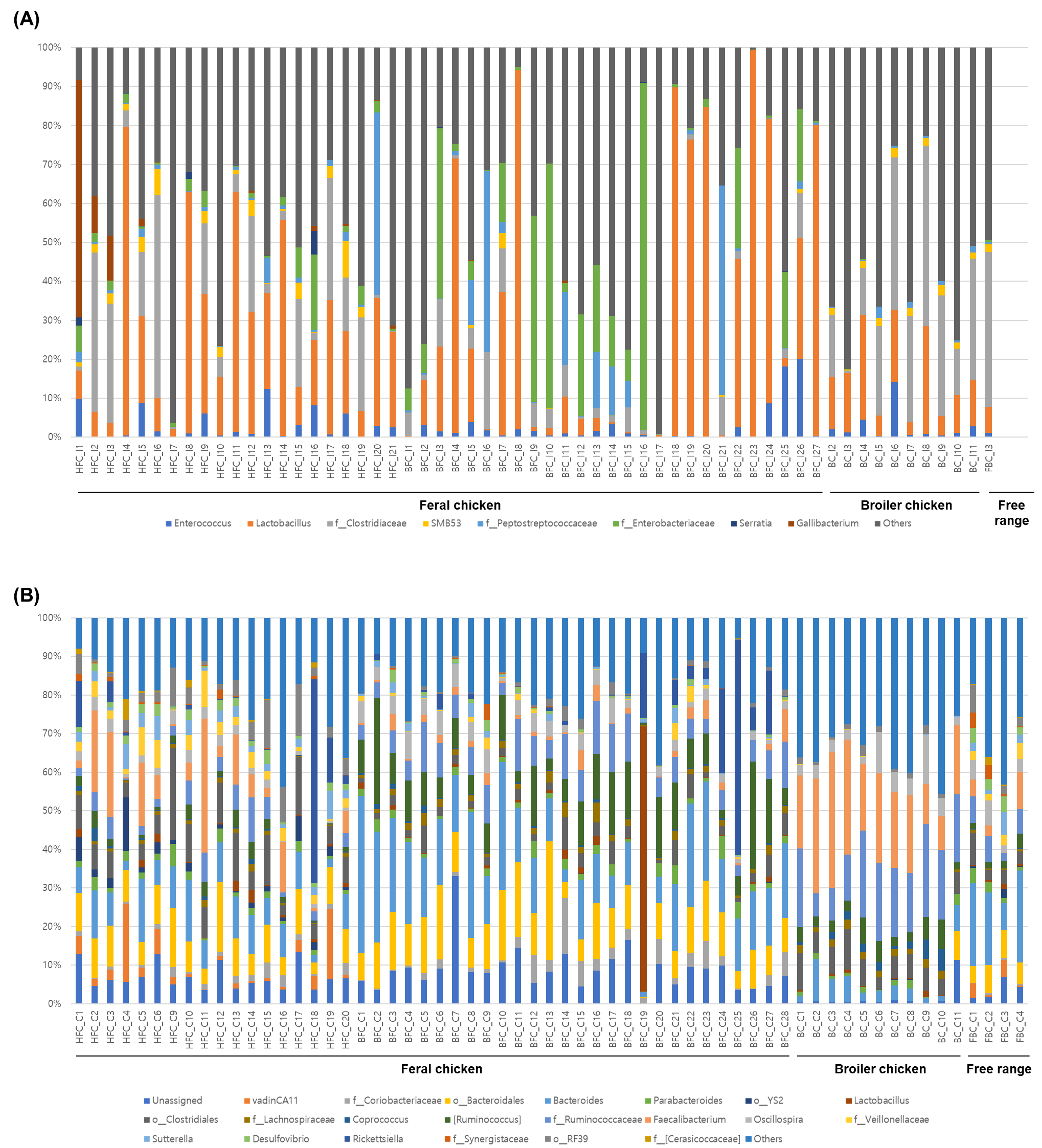
**

**Figure S3.** Relative abundance of major microbial taxa in the (A) ileum and (B) cecum of chickens sampled at the genus level.


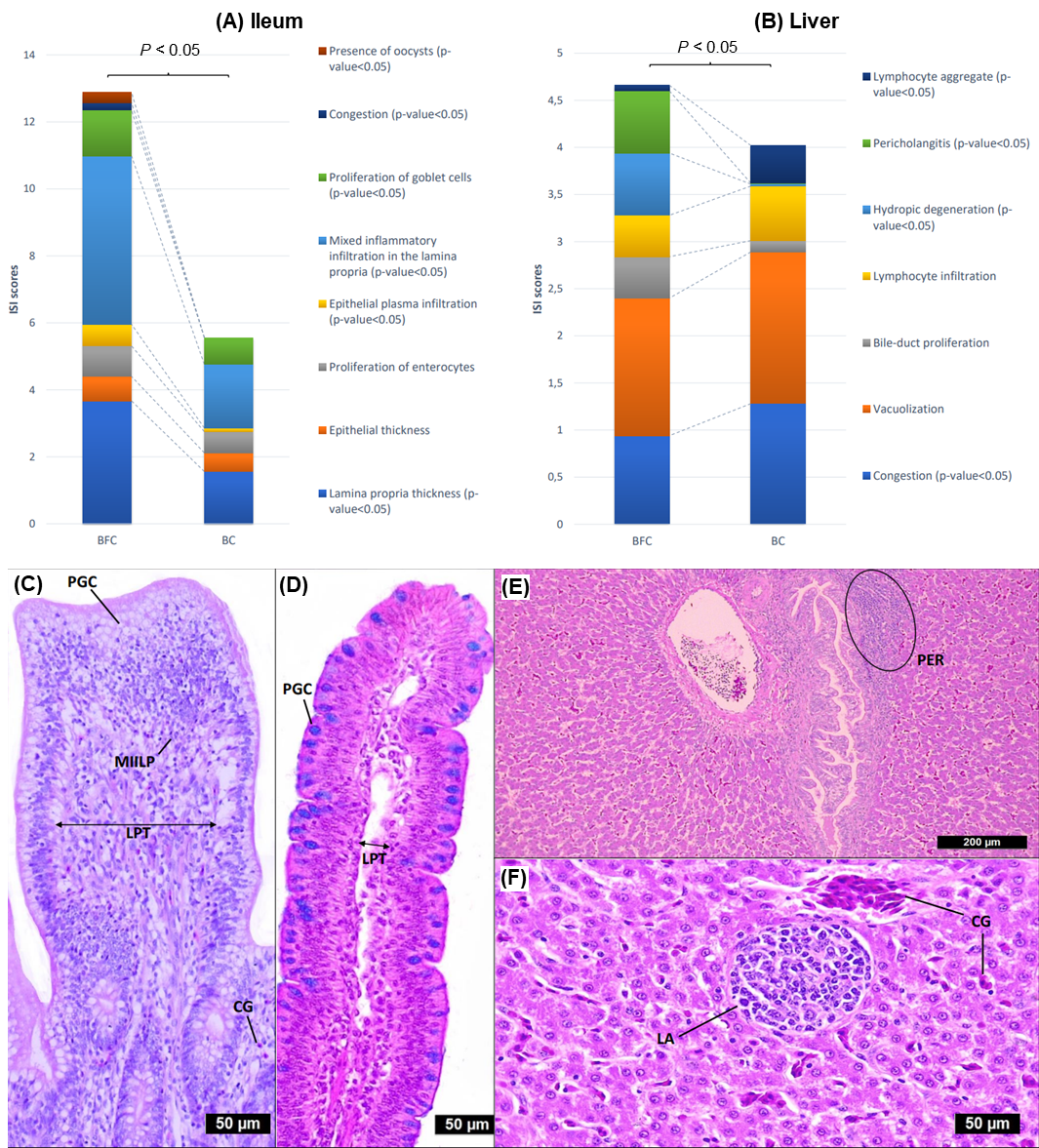


**Figure S4.** Metric evaluation of histological alterations in the (A) ileum and (B) liver based on the I See Inside (ISI) methodology. Representative microscopic images of (C, D) ileal mucosa and (E, F) liver from feral (C, E) and broiler (D, F) chickens stained with hematoxylin and eosin. PGC, pepsinogen C–positive cells; MIILP, mixed inflammatory infiltrate in the lamina propria; LPT, lamina propria T cells; CG, vascular congestion; PER, pericholangitis; LA, lymphoid aggregate.
